# Supplementary figures and images for: Copper binding leads to increased dynamics in the regulatory N-terminal domain of full-length human copper transporter ATP7B
Source: PLoS Comput Biol. 2022 Sep 7;18(9):e1010074. doi: 10.1371/journal.pcbi.1010074 (PMC9484656; doi:10.1371/journal.pcbi.1010074)

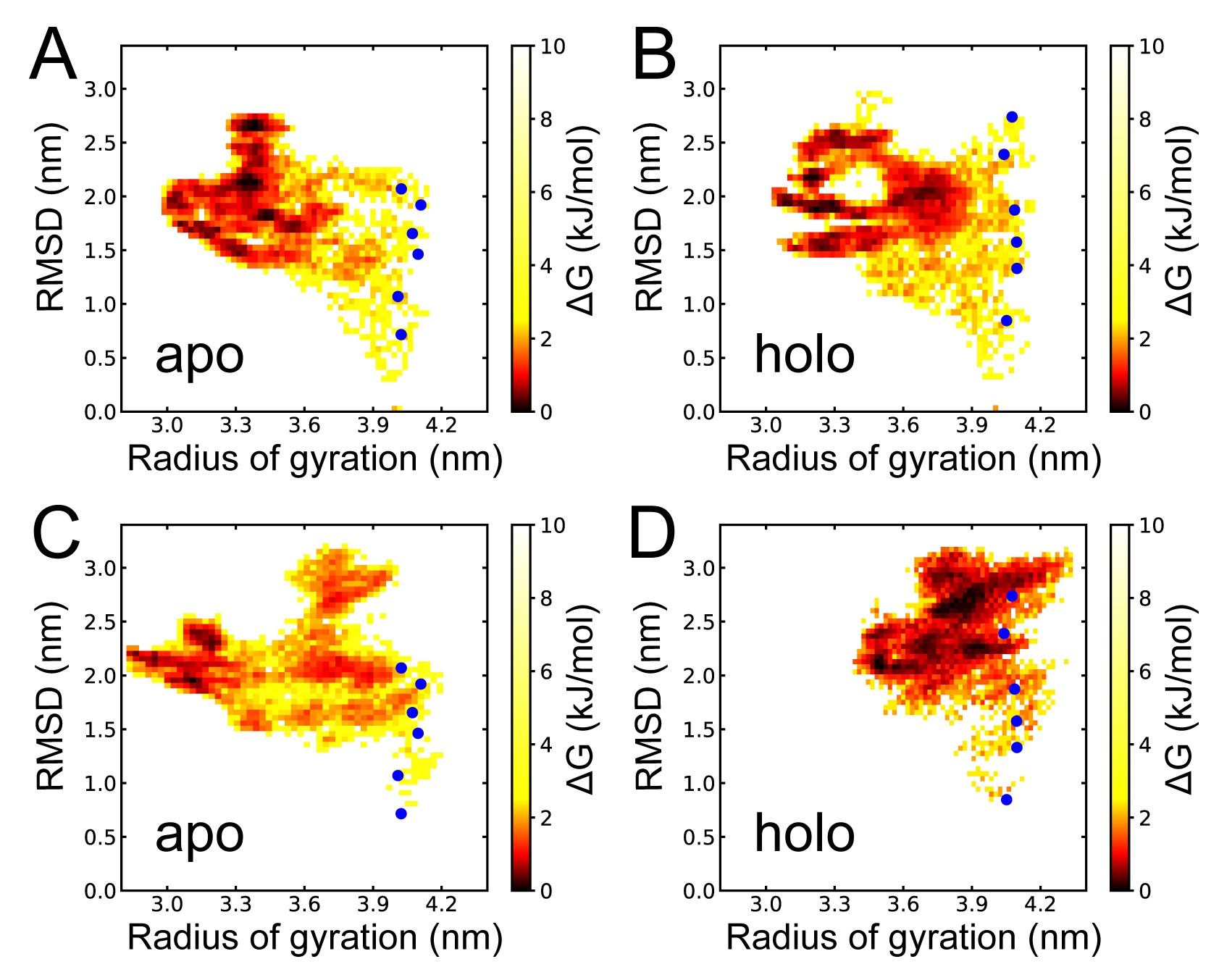

Supplement: S1 Fig — Free energy surfaces from the reA_apo (A), reA_holo (B), reB_apo (C) and reB_holo (D) simulations, defined by radius of gyration and RMSD to the N-terminal tail in the starting structure. Starting points for the reB simulations are marked with blue dots. (TIF) [file pcbi.1010074.s001.tif]

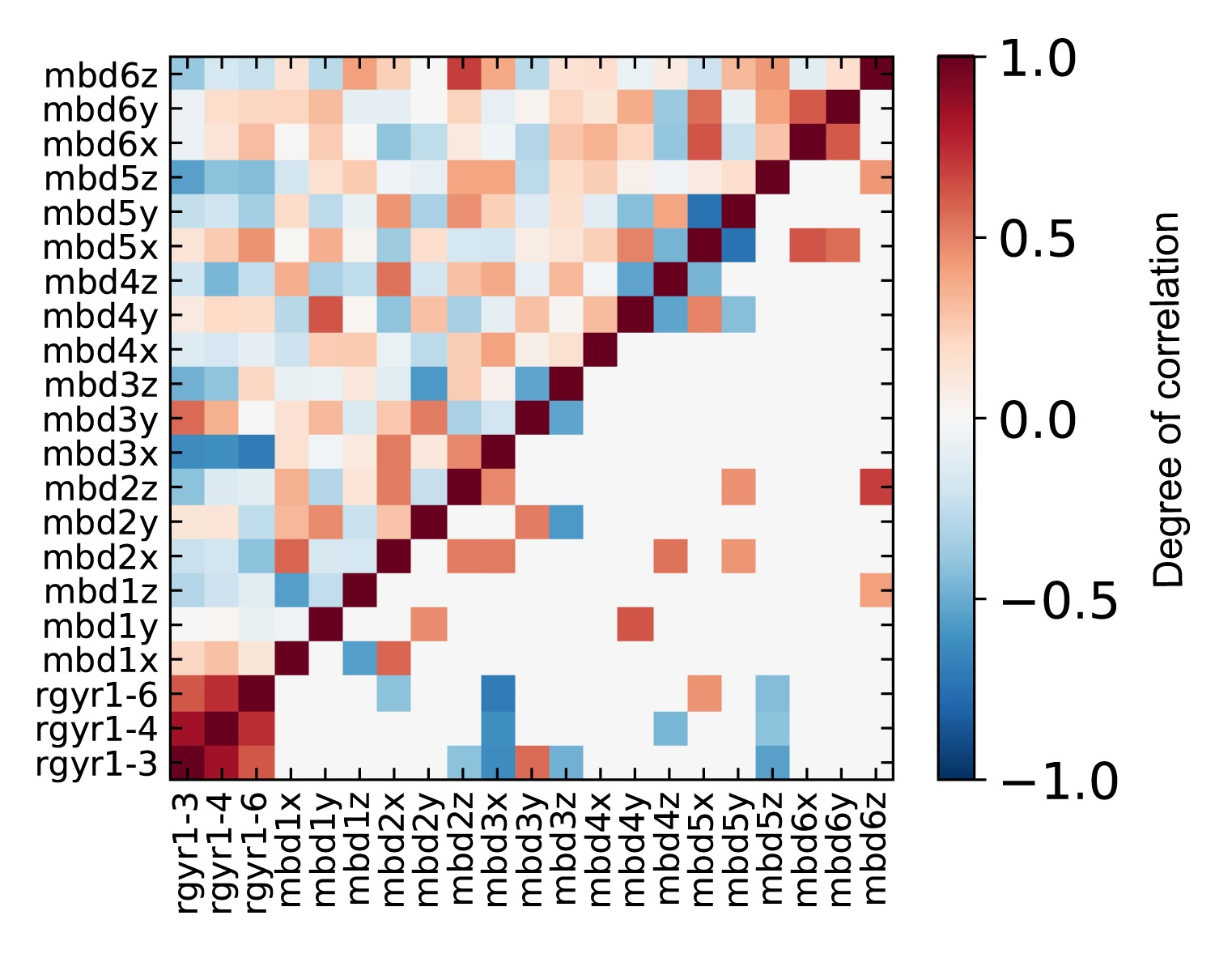

Supplement: S2 Fig — Cross-correlation matrix showing cross-correlations between the x, y and z positions of the MBDs and the radius of gyration of MBD1-3, MBD1-4 and MBD1-6. Red shows a positive correlation and blue means a negative correlation. The upper diagonal shows all correlations and the lower diagonal shows correlations with p < 0.05. (TIF) [file pcbi.1010074.s002.tif]

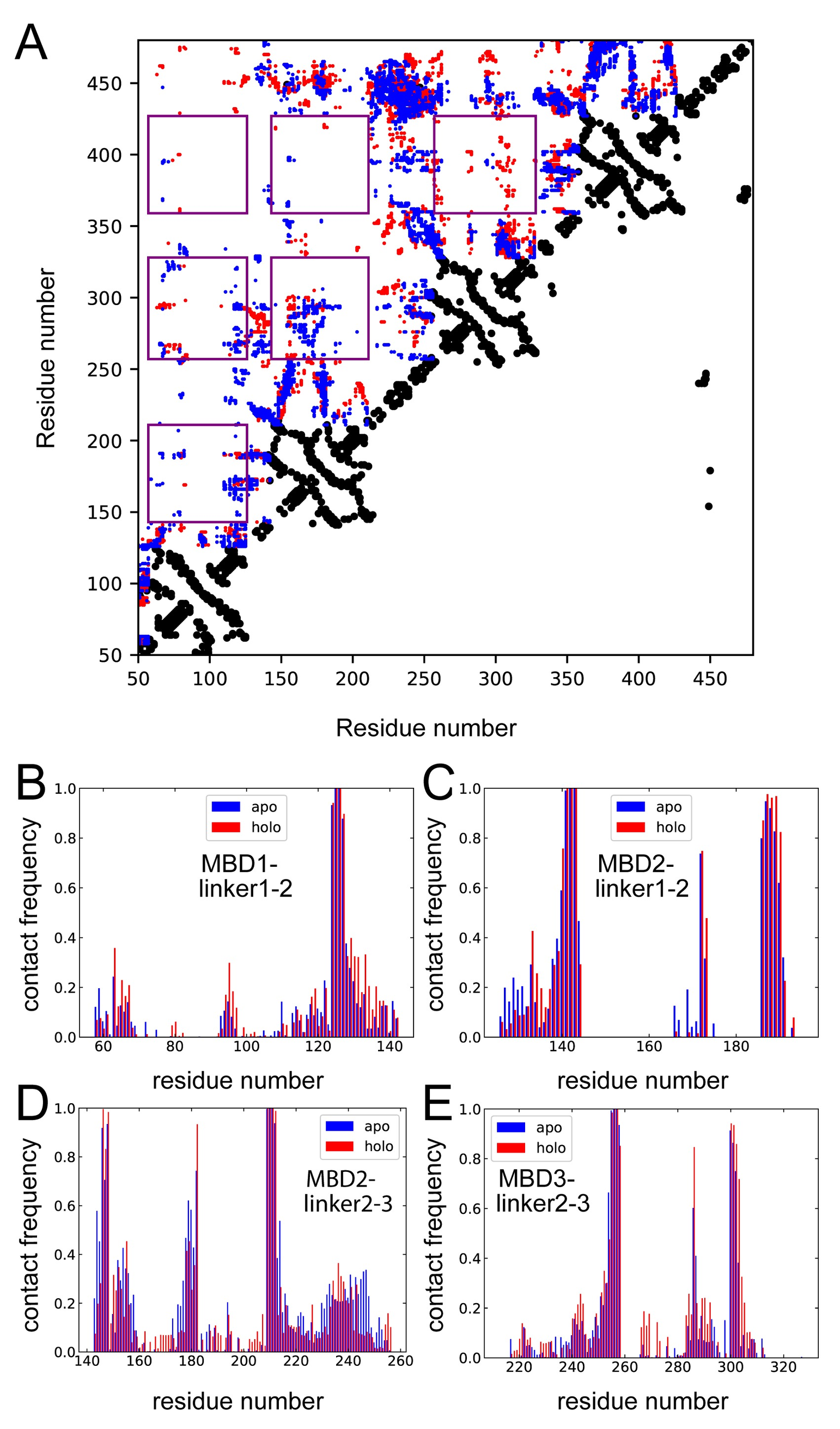

Supplement: S3 Fig — (A) Residue-residue contacts for MBD1-4 for holo (red) and apo (blue), with contacts between MBDs marked with purple boxes. Contact frequencies between residues in (B) MBD1 and the MBD1—MBD2 linker, (C) MBD2 and the MBD1-MBD2 linker, (D) MBD2 and the MBD2-MBD3 linker, and (E) MBD3 and the MBD2-MBD3 linker. (TIF) [file pcbi.1010074.s003.tif]

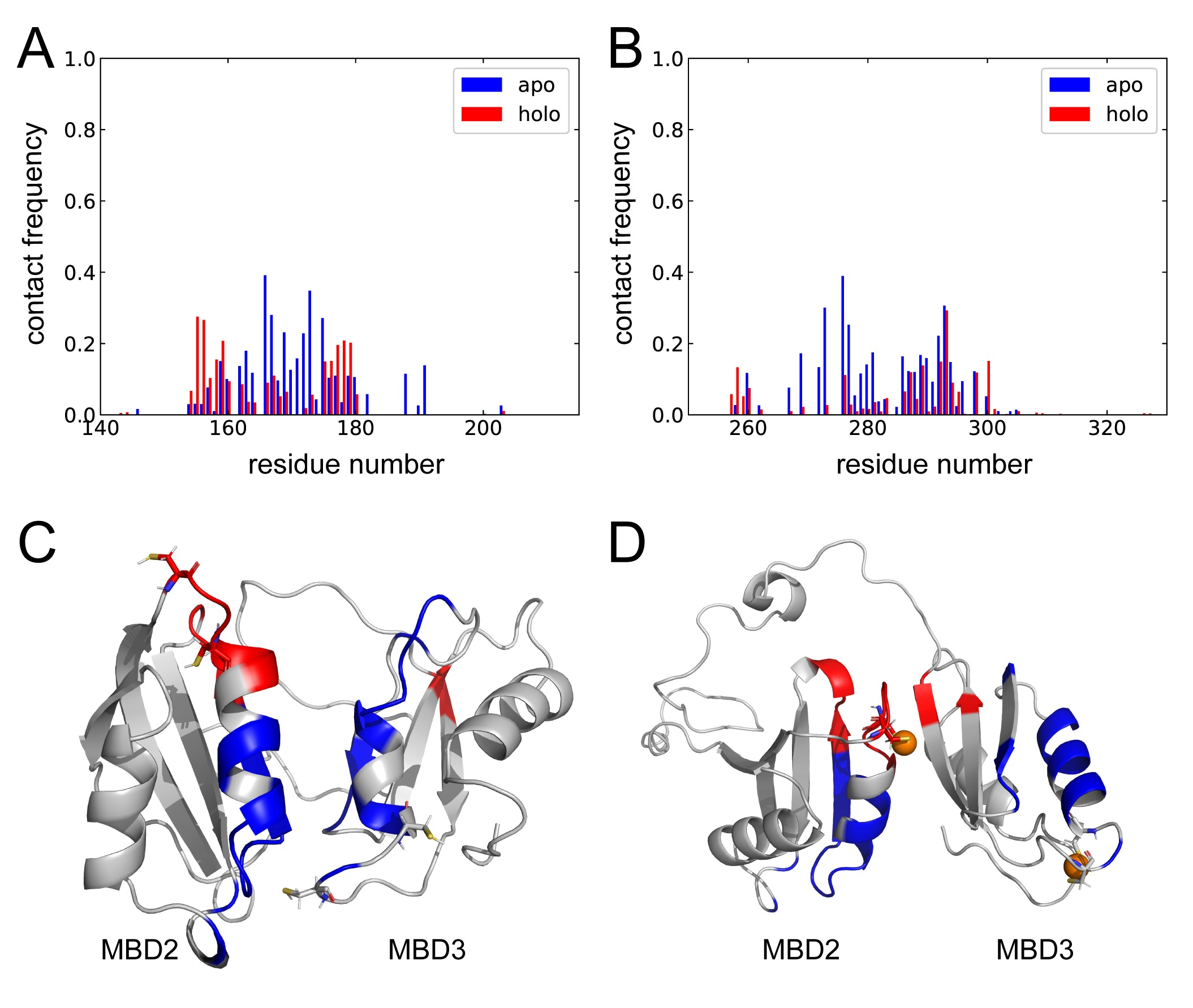

Supplement: S4 Fig — (A) Contact frequency per residue in MBD2 towards MBD3 and in (B) MBD3 towards MBD2. (C) Cartoon representation of MBD2 and MBD3 in the apo interaction position, with high contact frequency residues in blue (apo) and red (holo). The copper-binding cysteines are shown as sticks. (D) Cartoon representation of the holo interaction position, following the representation style used in (C), with copper shown as orange spheres. (TIF) [file pcbi.1010074.s004.tif]

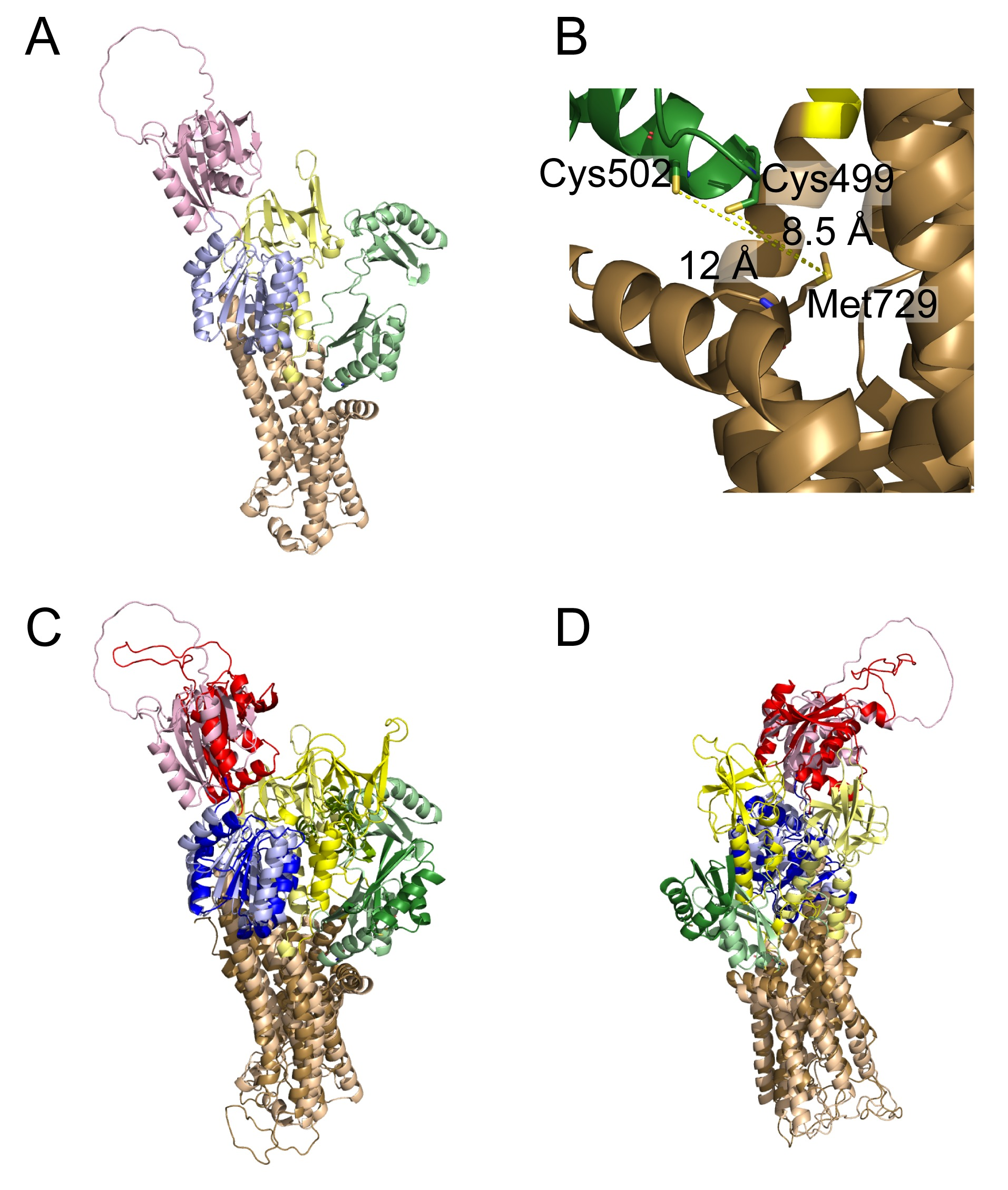

Supplement: S5 Fig — The M domain is shown in brown, the A domain in yellow, the P domain in blue, the N domain in red and the MBDs in green. (A) Overall view of the AlphaFold model, covering MBD5, MBD6 and the core domains. (B) Close-up view of the MBD5 copper-binding site and the copper-accepting Met729 in the membrane domain copper binding site, with cys-met sulfur distances in Å. (C) Alignment (by M and P domains) of the AlphaFold model with a representative structure from the holo simulations. The AlphaFold model is shown in lighter colors. (D) Alternate view of C), MBD6 not shown. (TIF) [file pcbi.1010074.s005.tif]

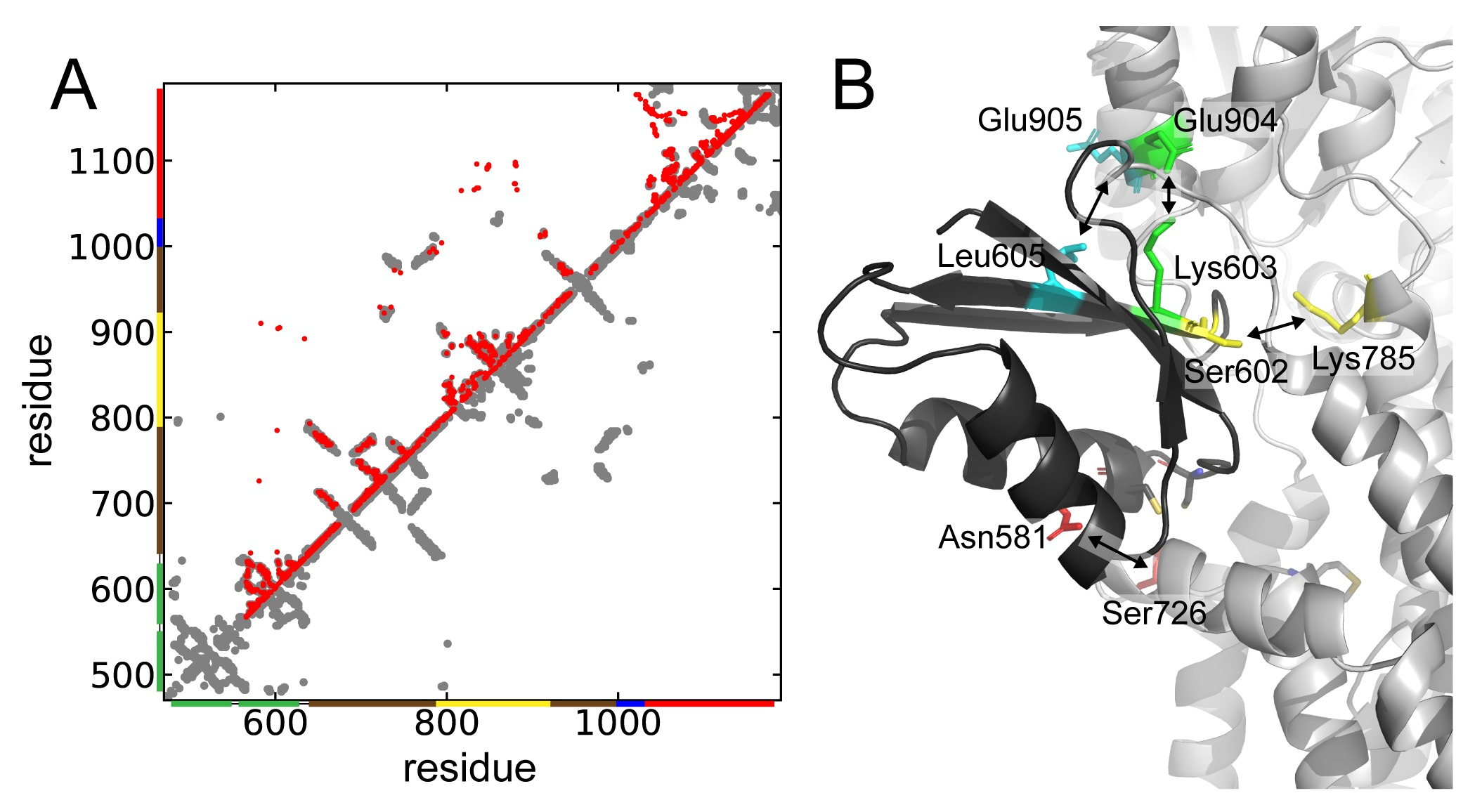

Supplement: S6 Fig — (A) Residue-residue contacts predicted by the EVcouplings server. Predicted contacts with a probability > 0.99 are shown as red dots, with contacts in the simulation starting structure in gray. (B) Position of MBD6 based on the coevolution contacts with MBD6 (black), Asn581-Ser726 (red), Ser602-Lys785 (yellow), Lys603-Glu904 (green), and Leu605-Glu905 (cyan). The rest of the protein is shown in gray. (TIF) [file pcbi.1010074.s006.tif]

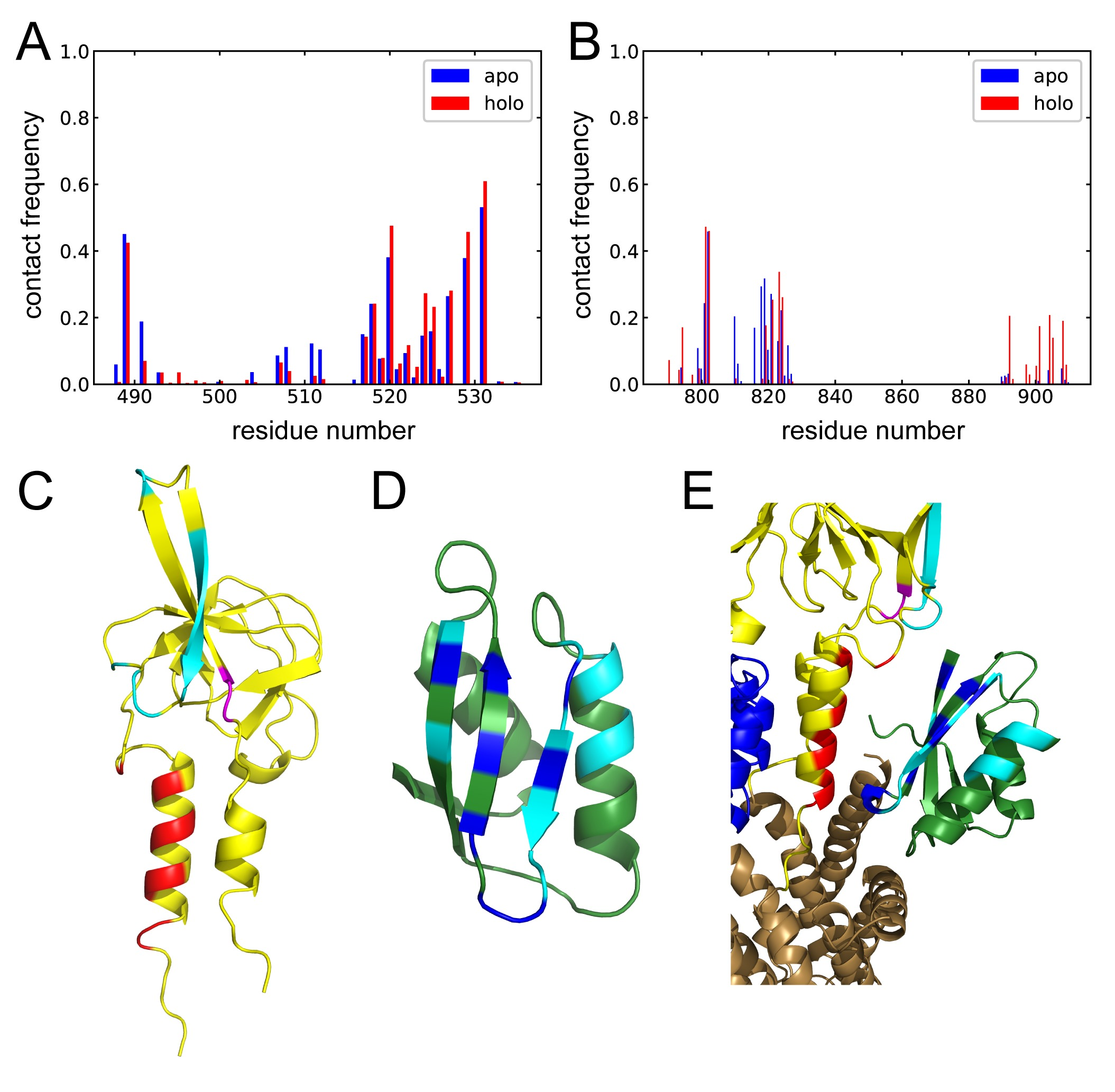

Supplement: S7 Fig — (A) MBD5 contact frequency against the A domain, and (B) A domain contact frequency against MBD5. (C) High contact frequency residues in the A domain are shown in magenta (cluster one, Thr801, Glu802), cyan (cluster two, residues in the range 810–826) and red (cluster 3, residues in the range 892–908), with the rest of the A domain in yellow. (D) High contact frequency residues in MBD5 are colored cyan (lower frequency) and blue (higher frequency), with the rest of MBD5 in green. (E) The A domain is shown in yellow and MBD5 is shown in green, with high contact frequency residues colored as in C) and D). The M domain is shown in brown and the P domain in blue. (TIF) [file pcbi.1010074.s007.tif]

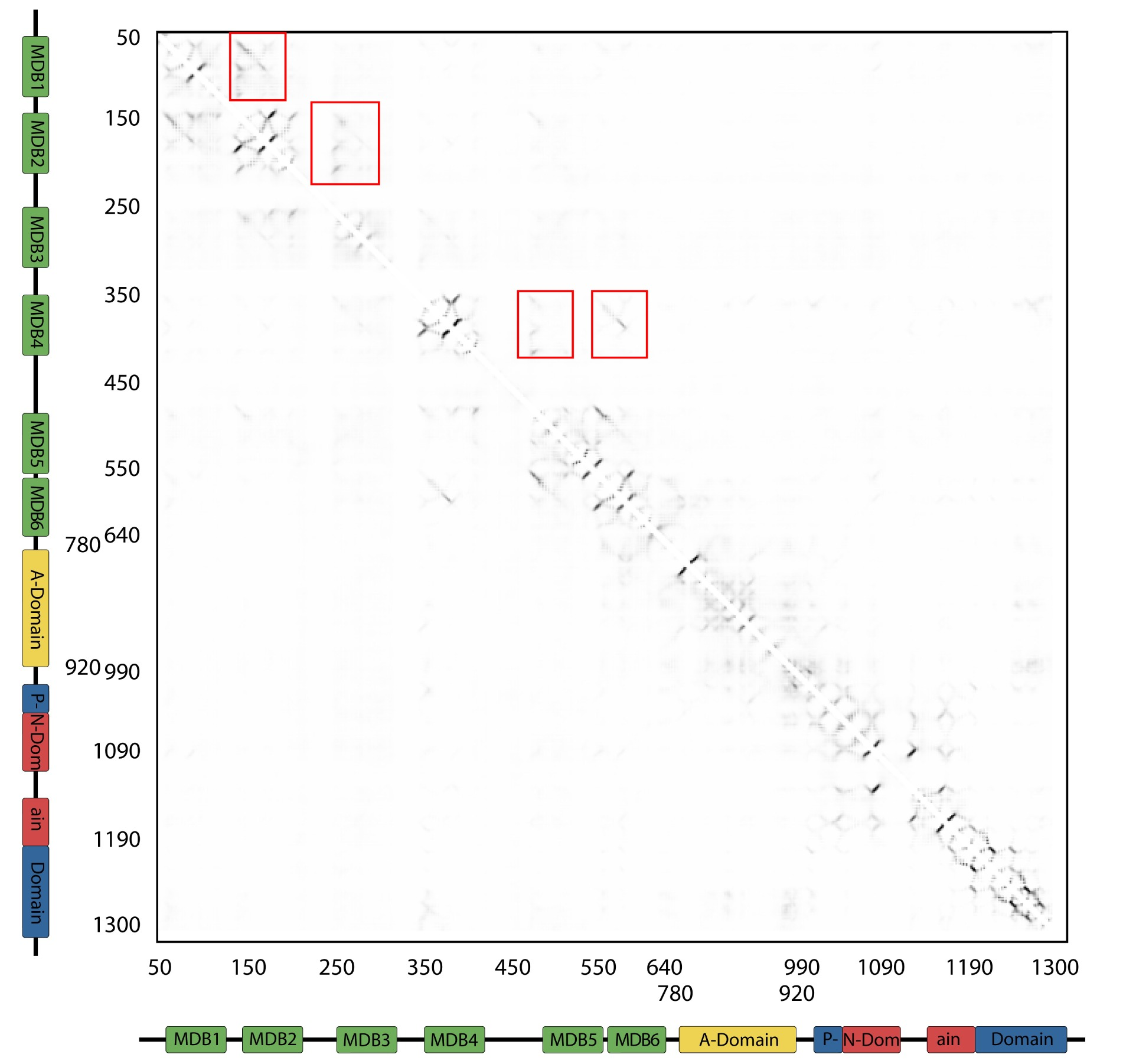

Supplement: S8 Fig — Analysis of evolutionary coupled amino acids was performed using the RaptorX server. Residues 1–49, 640–776, 922–994, 1313–1465 were omitted in the analysis (see Methods section). The resulting contact map shows predicted evolutionary pairs (black dots) within a Cβ-Cβ distance of 8 Å. Darker color indicates higher probability with the strongest identified interaction being in-between MBDs (red boxes). (TIF) [file pcbi.1010074.s008.tif]

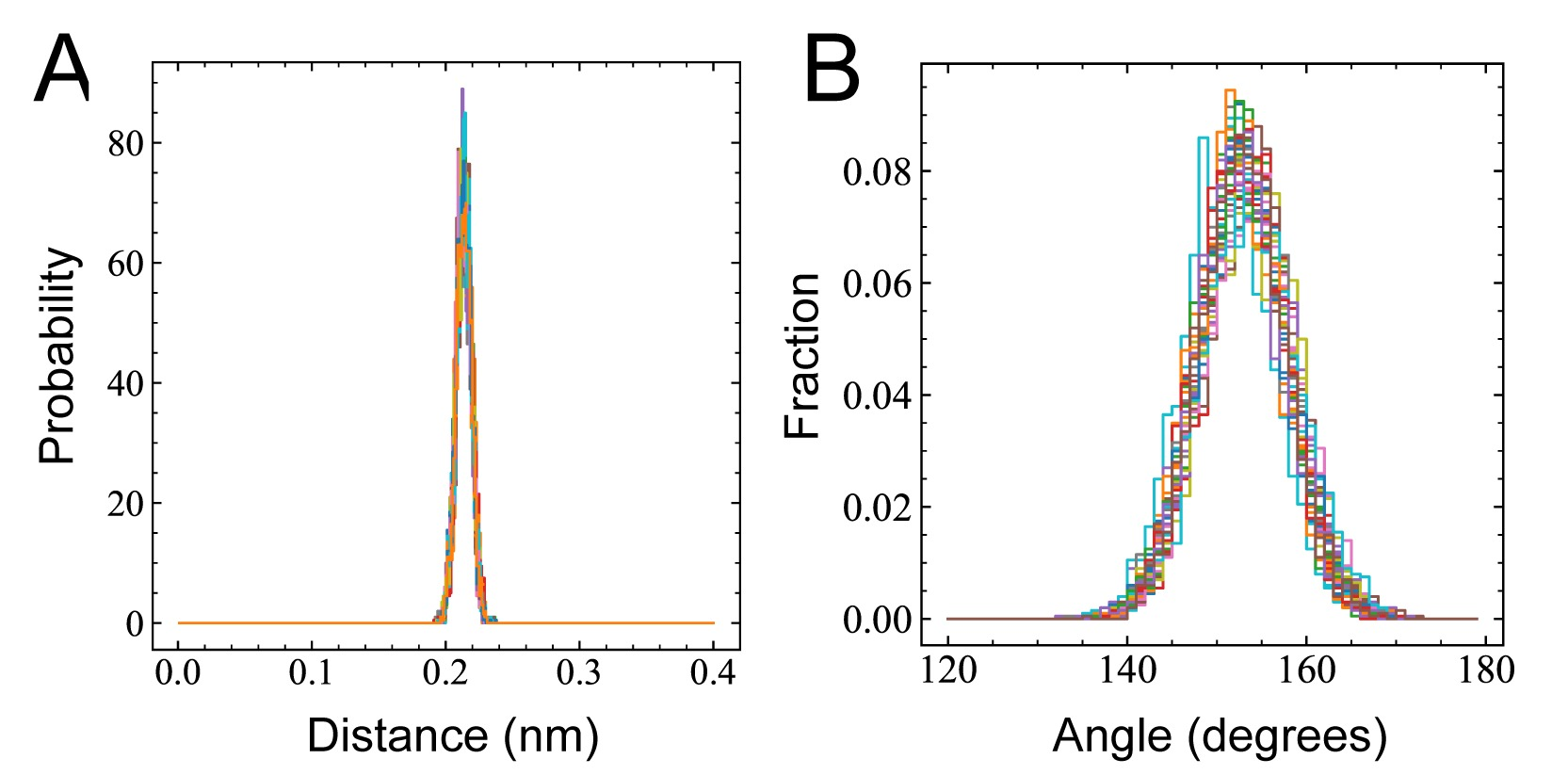

Supplement: S9 Fig — (A) Copper-sulfur distances for all copper-binding cysteines in the reA_holo simulations. (B) Sulfur-copper-sulfur angles for all MBDs in the reA_holo simulations. (TIF) [file pcbi.1010074.s009.tif]

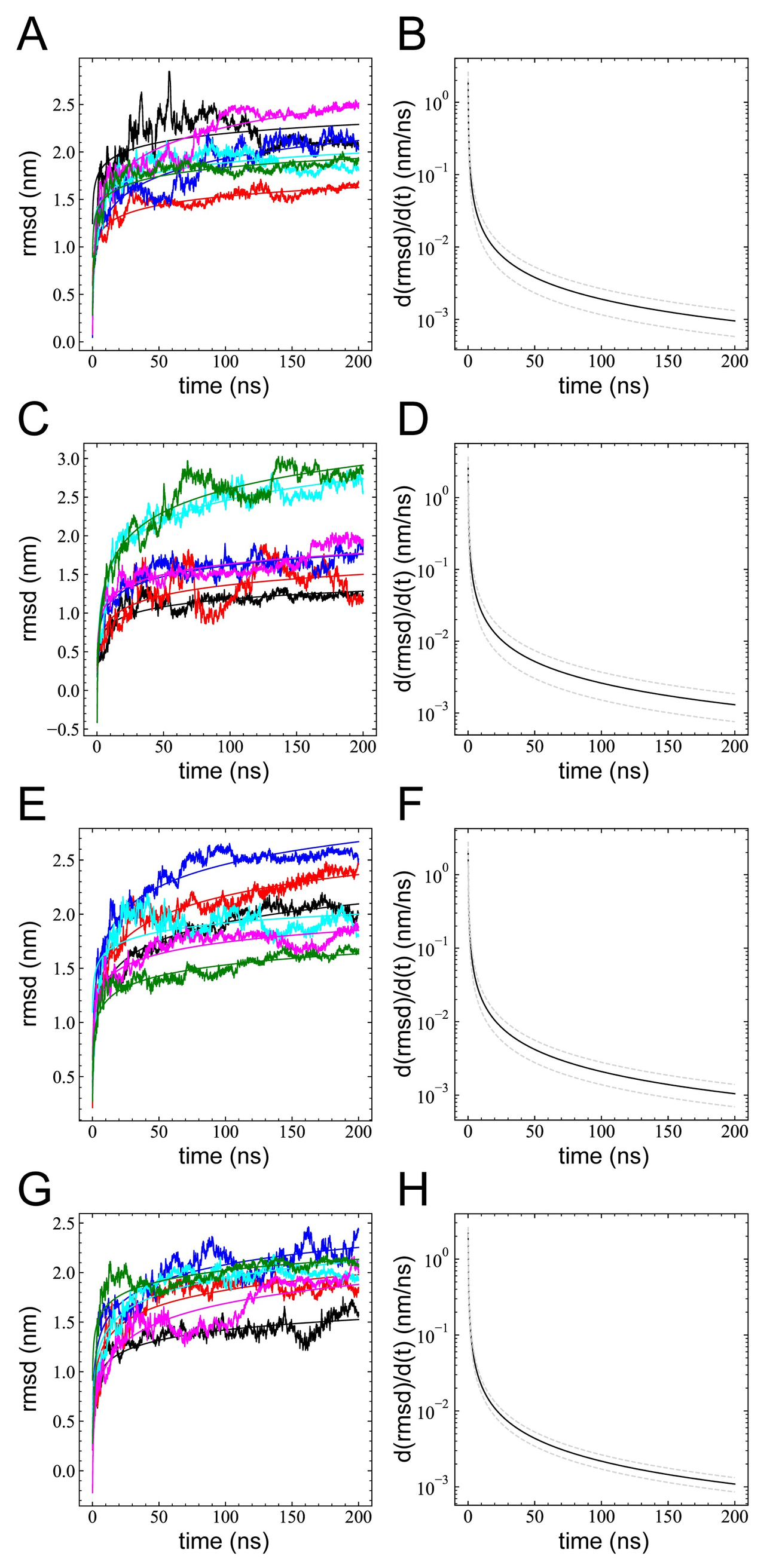

Supplement: S10 Fig — RMSD of the N-terminal domain and fitted logarithmic functions for A) reA_holo, C) reB_holo, E) reA_apo and G) reB_apo. Average derivative of the fitted logarithmic functions for B) reA_holo, D) reB_holo, F) reA_apo and H) reB_apo as solid black lines, with +/- standard deviation as dotted lines. (TIF) [file pcbi.1010074.s010.tif]

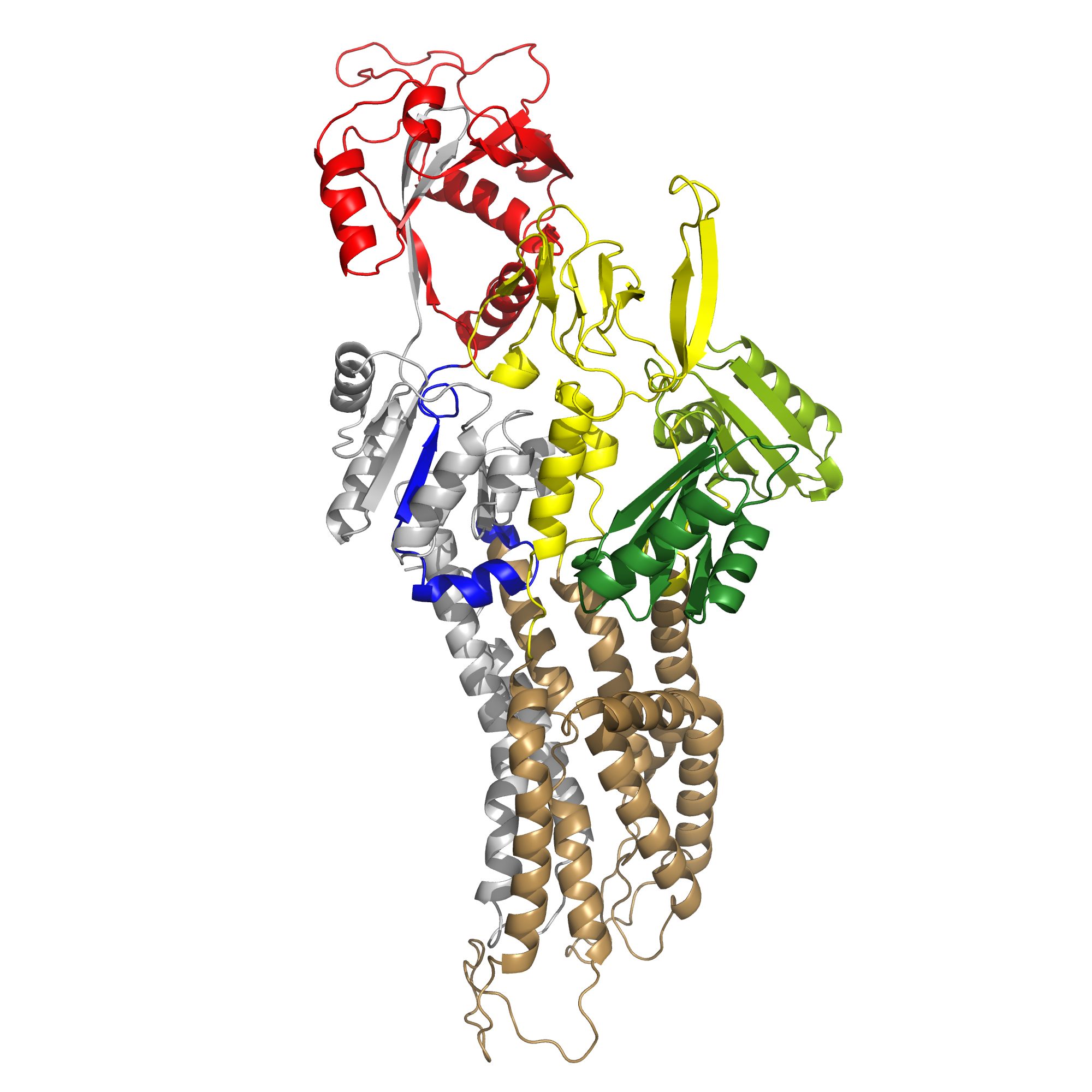

Supplement: S11 Fig — Residues that were not included are colored gray, the others are colored brown (M domain), yellow (A domain), blue (P domain), red (N domain), and green (MBD5 and MBD6). (TIF) [file pcbi.1010074.s011.tif]
