# Supplementary material for: Copper binding leads to increased dynamics in the regulatory N-terminal domain of full-length human copper transporter ATP7B
Source: PLoS Comput Biol. 2022 Sep 7;18(9):e1010074. doi: 10.1371/journal.pcbi.1010074 (PMC9484656; doi:10.1371/journal.pcbi.1010074)
Supplement: Attachment — Submitted filename: Response_to_reviewers.pdf [file pcbi.1010074.s012.pdf]

Reviewer #1: In this manuscript, MD simulations have been performed for the first time on the full N-terminal domain and ATP7B core domain. This is a very large system, and the conclusions drawn from this study are highly important, especially since till now no experiments have been performed on the whole ATP7B protein.
I therefore support publication, I have few minor suggestions:

We thank the Reviewer for these positive remarks on our work.

1. In the first paragraph of the results section, it will be nice if the authors can better explain how the homology model was created. Although it is explained in the methods, maybe also mention it here in brief, it will be easier to follow.

We agree and have now added the suggested explanation. Results section, p. 8 (blue text).

"The homology model was obtained from combining structural information from the bacterial homolog core protein, individual metal-binding ATP7B domains, NMR-guided modeling of N-terminal peptide, and modeling of the linker domain. The initial relative positioning of the metal-binding domains was obtained from experimental studies (see the Methods section for details)."

2. lines 182-183, I suggest to add the free energy values.

The free energy values refer to sampling frequency and should not be viewed as a biological property of the system. It is a means to measure statistical significance of the observed structural dynamics. Therefore, we think it is better not to explicitly state the free energy values in the text (the values are stated in the heatplot bars).

3. There is some experimental work that have been done on MBD1-3, MBD3-4, and MBD4-6, as a function of Atox1 and copper binding that support the conclusions of this manuscript, that was not mentioned here.

We have now added Zaccak et al., Int. J. Mol. Sci., 2020 and a Curr. Op. Struct. Biol. review (Magistrato et al., 2019) to further support the MBD interactions (Introduction section, p. 4 (blue text)). We have also added Qasem et al., Metallomics, 2019 (p. 5).

Reviewer #2: The article entitled ' Copper binding leads to increased dynamics in the regulatory N-terminal domain of full length human copper transported ATP7B' reports an extensive simulation study based on brute force MD and replica exchange simulations.

The simulations, based on an extensive modeling of the full length structure, show the influence of copper binding on the dynamics of the MBDs and suggest a possible mechanism of copper delivery from MBD5 to the transport region.
The article is interesting and has merit, but I think the authors need to clarify some points.

We thank the Reviewer for these positive remarks on our work.

In general, I think the main issue is that not many details about the replica exchange simulations are provided. 200 ns per replica for a structure of this size is sure not enough and no details are provided on the analysis of the convergence of the results. This is a very important point to understand he reliability of the results

We agree with the Reviewer and have now added Supplementary Figure 10 that shows that both the 1-6 reA replicas and the 1-6 reB replicas reach stabilization of the RMSD of the N-terminal tail. We also fitted logarithmic functions to the RMSD curves to show that the derivatives of these functions converge to 10^{-3} by the end of the simulations.

Given the size of the system, we used a replica exchange protocol as a means to enhance the sampling rather than attempting to determine absolute values of thermodynamical properties of the system, such as specific heat. Thus, for each condition (with and without Cu^{+}) we identify statistically significant structural differences based on 12 simulations starting either from different random seeds or from different extended conformations. We have now explained this in the manuscript (p. 8 and p. 22).

In addition, the free energies for individual replica exchange runs of the each holo and apo systems seem to be very different.

Indeed, there are differences between RE runs starting from the initial model (Suppl. Fig. 1 A,B) and starting from extended conformations (Suppl. Fig. 1 C,D). However, the trends for the apo systems to be more compact (low RGyr) compared to the more extended holo systems (high RGyr) are visible. We took great care in the statistical analyses to establish that the observed differences were significant ($p < 0.05$).

Many computational studies have been done to understand the effect of copper binding on the MBD and Atox1. These should be properly acknowledge. See for example Qasem Metallomics 2019 11 (7), 1288-1297, Magistrato Current Opinion in Structural Biology 2019 58, 26-33

We have now added the suggested references.

In addition, since the coordination geometry of Cu may be relevant to understand e the structural functional and dynamics properties of the systems I think the author should explicitly state which are the parameters used and the geometry.

We certainly agree and we have now added a description of the copper parameters in a specific subheading in the Methods section (p. 23-24).

How does the Cu coordination compare with B. T. Op't Holt Biochemistry, 2007, 46, 8816–8826 and to Qasem Metallomics 2019 11 (7), 1288-1297?

We used a similar copper coordination geometry as presented in Rodriguez-Granillo Biochemistry 2009 48, 5849-5863 and Rodriguez-Granillo J. Phys. Chem. 2010 114, 1836-1848, which is based on QM/MM simulations and result in larger S-Cu-S angles and a more linear bi-coordinated geometry than the tetrahedral coordination presented in Op't Holt Biochemistry 2007. This is in agreement with Qasem Metallomics 2019. We have now clarified this in the methods section (p. 21) and have added a new Supplemental Figure 9 that shows the resulting Cu-S distances and S-Cu-S angles.

Reviewer #3: The authors describe a molecular dynamics simulation of the chain of the cytosolic metal binding domains (MBDs) in the copper transporter ATP7B in the context of the whole protein. They show that in the copper-bound form, MBD2 and MBD3 experience greater freedom of motion resulting in a less compact arrangement of the MBD chain. This is consistent with a previously published model based on the NMR studies of the MBD1-6 fragment, which predicts transition of the MBD1-3 domain cluster to a more open

conformation, characterized by higher domain mobility, following copper transfer from the copper chaperone ATOX1 to the MBDs. MD simulations in the present work also show repositioning of MBD5, which is directly implicated in copper transfer to the copper-binding site in the transmembrane domain, in the holo-form of ATP7B.

While the general agreement between the computer simulation and the mechanistic models based on the previously published experimental data is important, the true value of molecular dynamics simulations is in providing non-trivial specific predictions that can be further tested experimentally, most commonly by site-directed mutagenesis combined with functional assays. This is where the paper falls a little short.

The authors identified some persistent interdomain residue contacts between the A-domain and MBD5 that show a change between the apo- and holo-forms, but there is very little specifics about the MBD1-3 group. One important question that remains unanswered is which interdomain residue contacts stabilize a more compact arrangement of the MBD1-3 domain cluster in the apo-form, and whether these contacts are disrupted by the copper binding in the simulation.

We have now added a more detailed analysis of interactions within the MBD1-3 domain group. Indeed, we identified significantly different contact frequencies between MBD2 and MBD3 in the apo and holo states. Interestingly, the interactions were different to those already reported in the literature and therefore constitute a new set of putative contact residues that can be experimentally tested. We have now added these results in two new Supplementary Figures (Suppl. Fig. 3 & 4) and new sections on interactions within the MBD1-3 domain group (p. 11-12, 16-17).

As suggested by the authors, there may be more than one set of such contacts, considering the highly dynamic domain arrangement. Yet, SAXS (Yu et al, 2017, ref. 32) shows a distinct shape of the conformational space occupied by the MBD1-3 cluster in the apo-form, indicating well-defined domain-domain interactions. Perhaps, conducting MD simulation with copper bound only to the MBDs 1-3, as opposed to all six MBDs, will provide some additional insight.

We agree and have now added more detailed analysis of the MBD1-3 group, as explained above. We found copper-dependent differences in interactions between MBD2 and MBD3 in a previously unreported arrangement. Although we agree that variation in the levels of copper binding in the MBDs is highly interesting (now mentioned on p. 16-17) - because of the complexity of the system, we have chosen to compare apo and holo states in this work. Reaching the same level of statistical significance for an additional state will require a major addition to an already extensive set of simulations that pushes the limits of what types of structural complexity can be assessed by MD simulations today.
